# Supplementary material for: Systematic comparison of monoclonal versus polyclonal antibodies for mapping histone modifications by ChIP-seq
Source: Epigenetics Chromatin. 2016 Nov 4;9:49. doi: 10.1186/s13072-016-0100-6 (PMC5097419; doi:10.1186/s13072-016-0100-6)

Figure S1. Reads aligning to annotated open and closed chromatin. The location of reads of different insert sizes from our K562 WCE data was compared to the ENCODE annotation of the genome using BEDTools [1]. Reads with smaller insert sizes, indicating that they originate from smaller DNA fragments, mapped to transcription start sites (open chromatin) at a higher rate than those of longer insert sizes. An opposite relationship was oboserved for closed chromatin. Note, however, that the Y axis is truncated to show this effect and the change in the percentage of reads tracking to repressed regions is small. Reads below and above the displayed insert sizes were excluded due to low read coverage and associate noise.

Figure S2: Read coverage across the genome. Images of Tiled Data Files (TDFs) generated by the IGV browser [2, 3] displaying the density tracks of reads aligned across the genome. The tracks show the correspondence in read coverage in monoclonal and polyclonal antibodies (from ENCODE and Mouse ENCODE) over representative genomic loci. **A-C** GM12878; **D-F** mES. **A.** Chromosome 19:41,792,290-41,912,756 (about 120Kb) for H3K4me1, H3K4me3 (two monoclonal lots) and H3K27ac. **B.** Chromosome 20:61,503,810-62,032,504 (about 500Kb) for H3K27me3. **C.** Chromosome (about 250Kb) for H3K9me3. **D.** Chromosome 6:125,035,929-125,153,426 (about 120Kb) for H3K4me1, H3K4me3 (two monoclonal lots) and H3K27ac. **E.** Chromosome 6:51,949,208-52,399,454 (about 450Kb) for H3K27me3. **F.** Chromosome 8:65,216,638-65,604,437 (about 390Kb) for H3K9me3.

Figure S3: Saturation curve showing the number of bases called as being in peaks as a function of sequencing depth for H3K9me3 and H3K27me3. The final dataset of the merged technical replicates was randomly down sampled to 20 different read depths and peaks were called in each dataset using HOMER. For these datasets, curves do not show the expected pattern and do not appear to reach saturation.

Figure S4: **Mapping of peaks and reads to canonical chromatin regions of the genome as defined by the ENCODE mappings.** These plots display the percentage of reads that map to each canonical genome region. The canonical genome regions were defined by the combined ENCODE mapping and are abbreviated as follows: CTCF-enriched elements (*CTCF*), promoter flanking regions (*PF*), transcription start sites (*TSS*), transcribed regions (*T*), enhancers (*E*), weak enhancers (*WE*), and repressed regions (*R*). Reads were normalized by random downsampling.

Figure S5. Correlation between monoclonal and polyclonal antibodies across the genome. Scatter plots (Loglog) show counts of reads per bin in non-overlapping 2000 bp bins tiled throughout the genome in replicates of the monoclonal antibody (**left**), the polyclonal antibody (**right**), and polyclonal antibody versus monoclonal antibody (**center**). The reads for each dataset were normalized by **A.** insert size or by **B.** random downsampling.

Figure S6: Experimental quality control. While we generated four technical replicates of the H3K27me3 polyclonal antibody, one of the replicates (Replicate number 4) did not pass our quality control. Points on the graph represent the number of reads falling into each variable-sized bin defined by the canonical chromatin regions of the genome as defined by the ENCODE. **A.** A comparison of Replicate 3 to the summed read counts of Replicates 1 and 2. This is in line with our expectations. **B.** The same comparison to Replicates 1 and 2, this time using Replicate 4. Here we see that Replicate 4 has a systematically reduced read count in transcription start sites. No other classes of genomic regions were shifted. We concluded that Replicates 4 did not pass our quality control.

Figure S7: Validation of the polyclonal antibody targeting H3K4me1 by peptide array. The details are presented in the same manner as the publically available validations of the other antibodies we used in the study (**Table 1**), and include the details of the antibody and validation protocol, the row signal of the

peptide array (**bottom left**); reactivity with the synthetic peptides on the array (**top right**), and the cross reactivity other modifications of the same peptide.

Supplemental References

1. Quinlan AR: **BEDTools: The Swiss-Army Tool for Genome Feature Analysis**. *Current protocols in bioinformatics / editoral board, Andreas D Baxevanis [et al]* 2014, **47**:11 12 11-11 12 34.
2. Robinson JT, Thorvaldsdottir H, Winckler W, Guttman M, Lander ES, Getz G, Mesirov JP: **Integrative genomics viewer**. *Nature biotechnology* 2011, **29**(1):24-26.
3. Thorvaldsdottir H, Robinson JT, Mesirov JP: **Integrative Genomics Viewer (IGV): high-performance genomics data visualization and exploration**. *Brief Bioinform* 2013, **14**(2):178-192.

Figure S1.

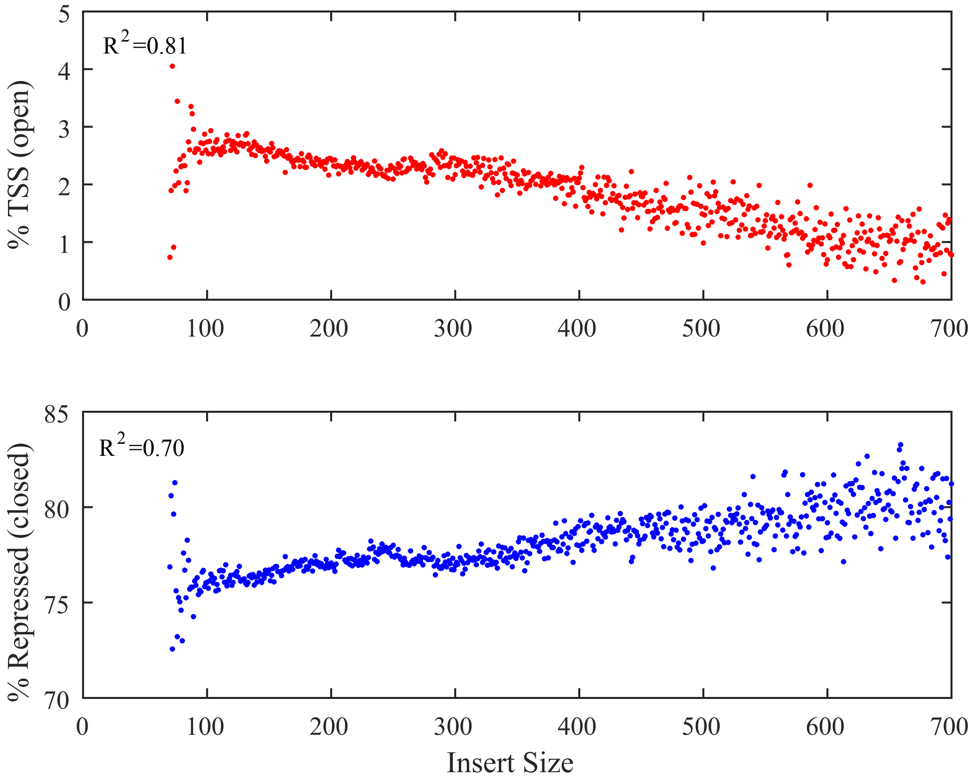

R^2=0.81
% TSS (open)
5
4
3
2
1
0
0
100
200
300
400
500
600
700
R^2=0.70
% Repressed (closed)
85
80
75
70
0
100
200
300
400
500
600
700
Insert Size

Figure S2.

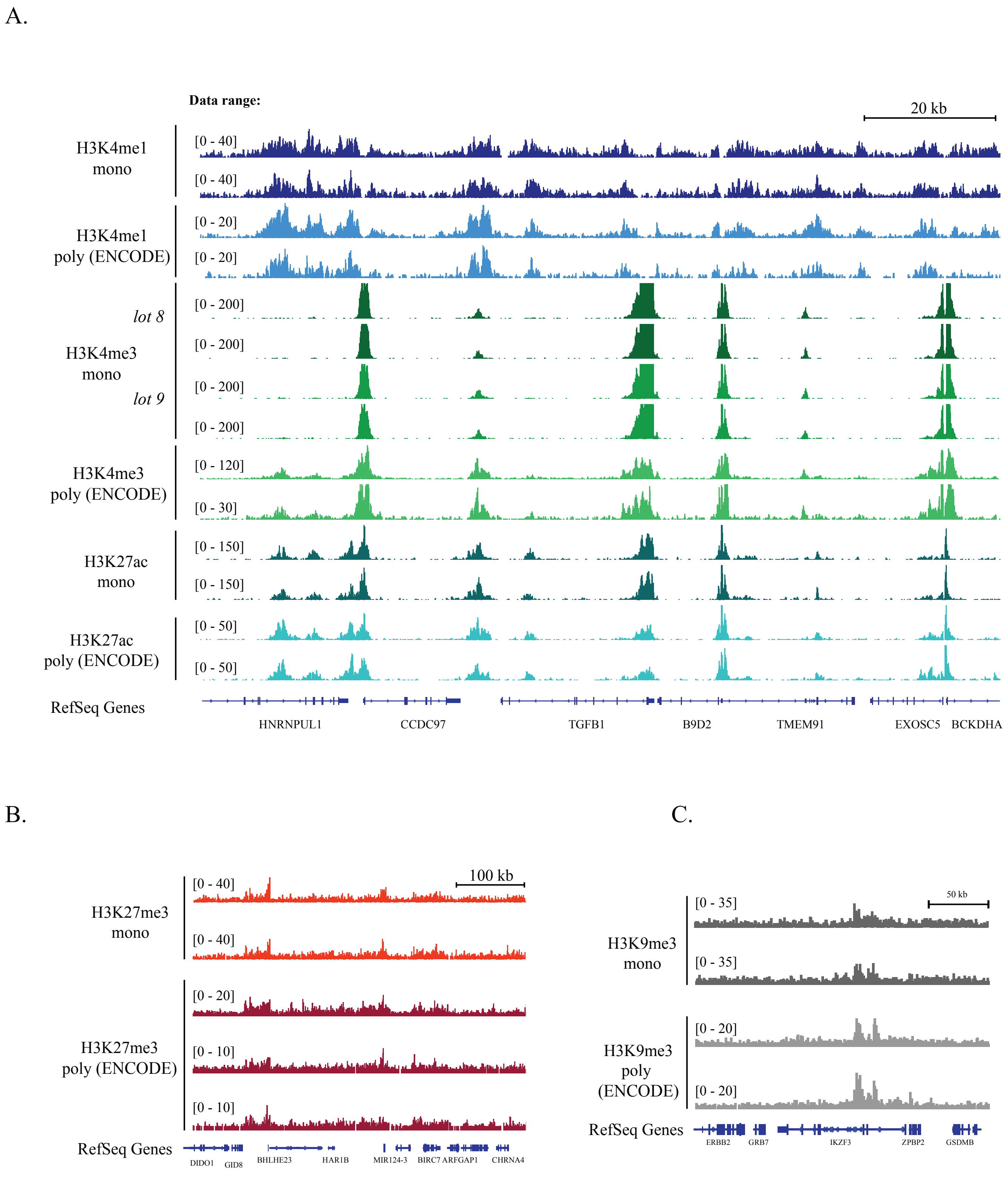
A.
Data range:
20 kb
H3K4me1
mono
[0 - 40]
[0 - 40]
H3K4me1
poly (ENCODE)
[0 - 20]
[0 - 20]
lot 8
H3K4me3
mono
lot 9
[0 - 200]
[0 - 200]
[0 - 200]
[0 - 200]
H3K4me3
poly (ENCODE)
[0 - 120]
[0 - 30]
H3K27ac
mono
[0 - 150]
[0 - 150]
H3K27ac
poly (ENCODE)
[0 - 50]
[0 - 50]
RefSeq Genes
HNRNPUL1
CCDC97
TGFB1
B9D2
TMEM91
EXOSC5
BCKDHA
B.
H3K27me3
mono
[0 - 40]
100 kb
[0 - 40]
H3K27me3
poly (ENCODE)
[0 - 20]
[0 - 10]
[0 - 10]
RefSeq Genes
DIDO1
GID8
BHLHE23
HAR1B
MIR124-3
BIRC7
ARFGAP1
CHRNA4
C.
H3K9me3
mono
[0 - 35]
50 kb
[0 - 35]
H3K9me3
poly
(ENCODE)
[0 - 20]
[0 - 20]
RefSeq Genes
ERBB2
GRB7
IKZF3
ZPBP2
GSDMB

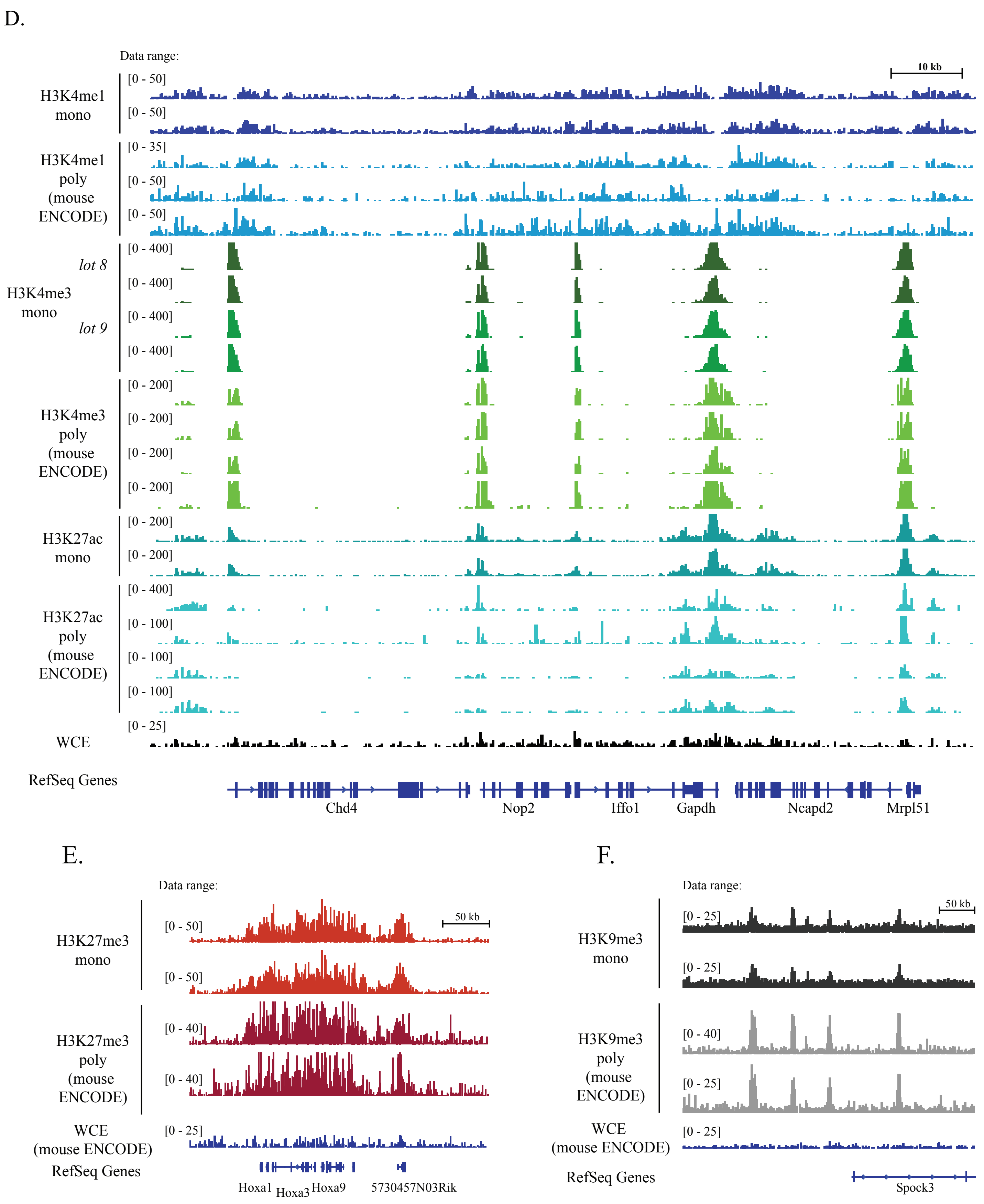

D.
Data range:
10 kb
H3K4me1 mono
[0 - 50]
[0 - 50]
H3K4me1 poly (mouse ENCODE)
[0 - 35]
[0 - 50]
[0 - 50]
H3K4me3 mono
lot 8
lot 9
[0 - 400]
[0 - 400]
[0 - 400]
[0 - 400]
H3K4me3 poly (mouse ENCODE)
[0 - 200]
[0 - 200]
[0 - 200]
[0 - 200]
H3K27ac mono
[0 - 200]
[0 - 200]
H3K27ac poly (mouse ENCODE)
[0 - 400]
[0 - 100]
[0 - 100]
[0 - 100]
WCE
[0 - 25]
RefSeq Genes
Chd4
Nop2
Iffo1
Gapdh
Ncapd2
Mrpl51
E.
Data range:
50 kb
H3K27me3 mono
[0 - 50]
[0 - 50]
H3K27me3 poly (mouse ENCODE)
[0 - 40]
[0 - 40]
WCE (mouse ENCODE)
[0 - 25]
RefSeq Genes
Hoxa1
Hoxa3
Hoxa9
5730457N03Rik
F.
Data range:
50 kb
H3K9me3 mono
[0 - 25]
[0 - 25]
H3K9me3 poly (mouse ENCODE)
[0 - 40]
[0 - 25]
WCE (mouse ENCODE)
[0 - 25]
RefSeq Genes
Spock3

Figure S3

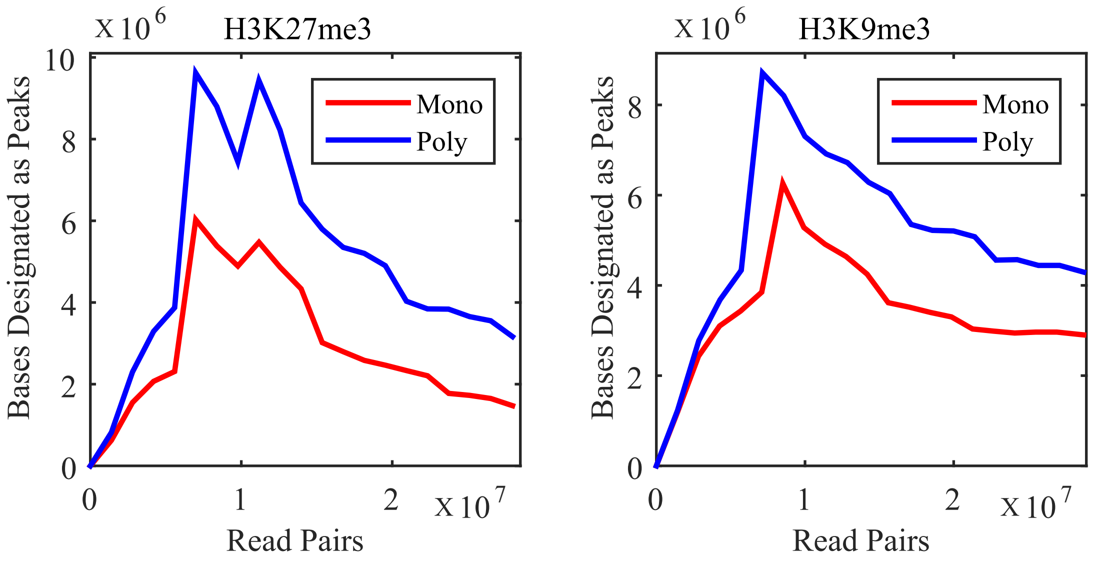
x 10^6
H3K27me3
10
8
6
4
2
0
Bases Designated as Peaks
Mono
Poly
0
1
2
x 10^7
Read Pairs
x 10^6
H3K9me3
8
6
4
2
0
Bases Designated as Peaks
Mono
Poly
0
1
2
x 10^7
Read Pairs

Figure S4.

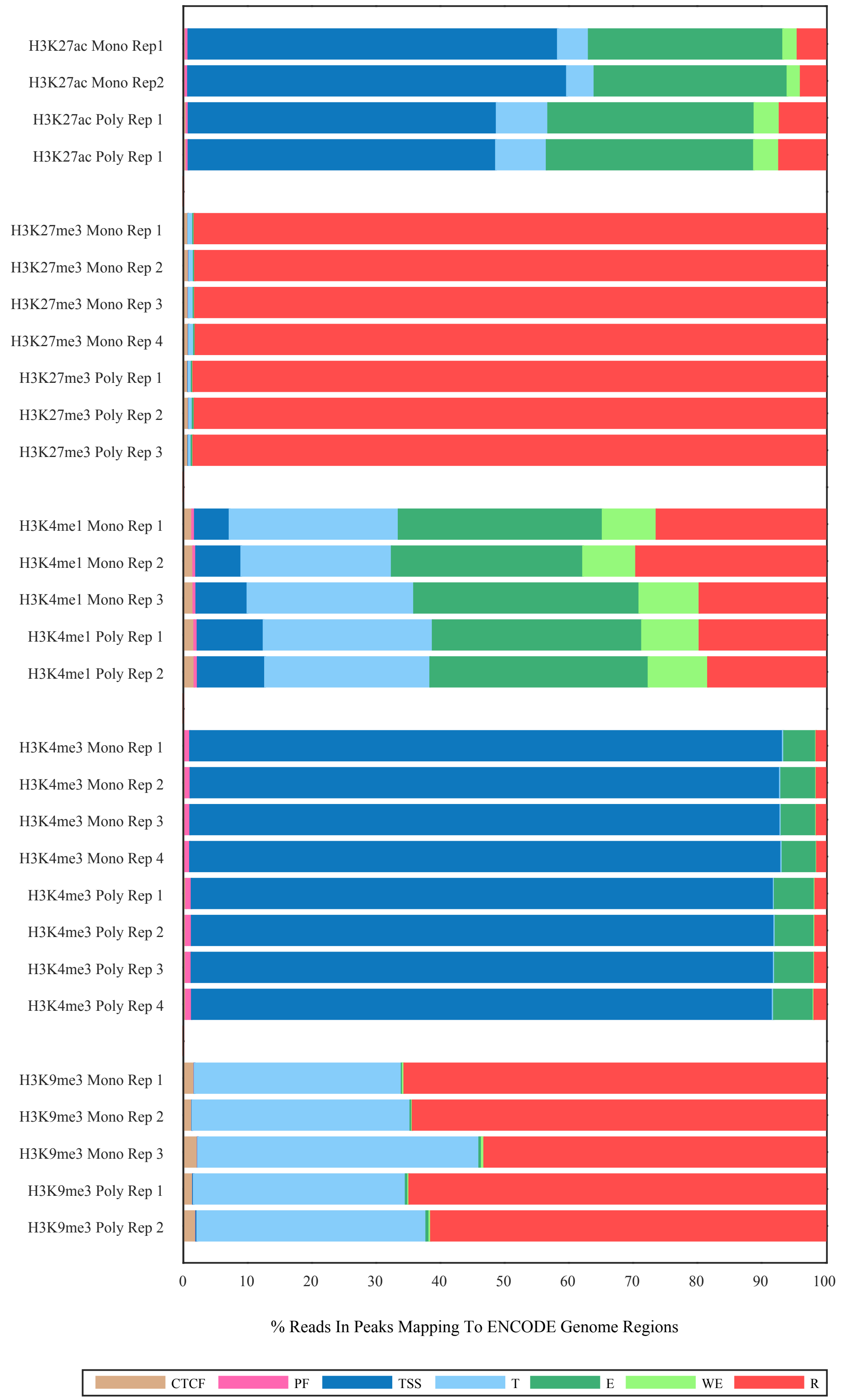
H3K27ac Mono Rep1
H3K27ac Mono Rep2
H3K27ac Poly Rep 1
H3K27ac Poly Rep 1
H3K27me3 Mono Rep 1
H3K27me3 Mono Rep 2
H3K27me3 Mono Rep 3
H3K27me3 Mono Rep 4
H3K27me3 Poly Rep 1
H3K27me3 Poly Rep 2
H3K27me3 Poly Rep 3
H3K4me1 Mono Rep 1
H3K4me1 Mono Rep 2
H3K4me1 Mono Rep 3
H3K4me1 Poly Rep 1
H3K4me1 Poly Rep 2
H3K4me3 Mono Rep 1
H3K4me3 Mono Rep 2
H3K4me3 Mono Rep 3
H3K4me3 Mono Rep 4
H3K4me3 Poly Rep 1
H3K4me3 Poly Rep 2
H3K4me3 Poly Rep 3
H3K4me3 Poly Rep 4
H3K9me3 Mono Rep 1
H3K9me3 Mono Rep 2
H3K9me3 Mono Rep 3
H3K9me3 Poly Rep 1
H3K9me3 Poly Rep 2
0 10 20 30 40 50 60 70 80 90 100
% Reads In Peaks Mapping To ENCODE Genome Regions
CTCF PF TSS T E WE R

Figure S5.

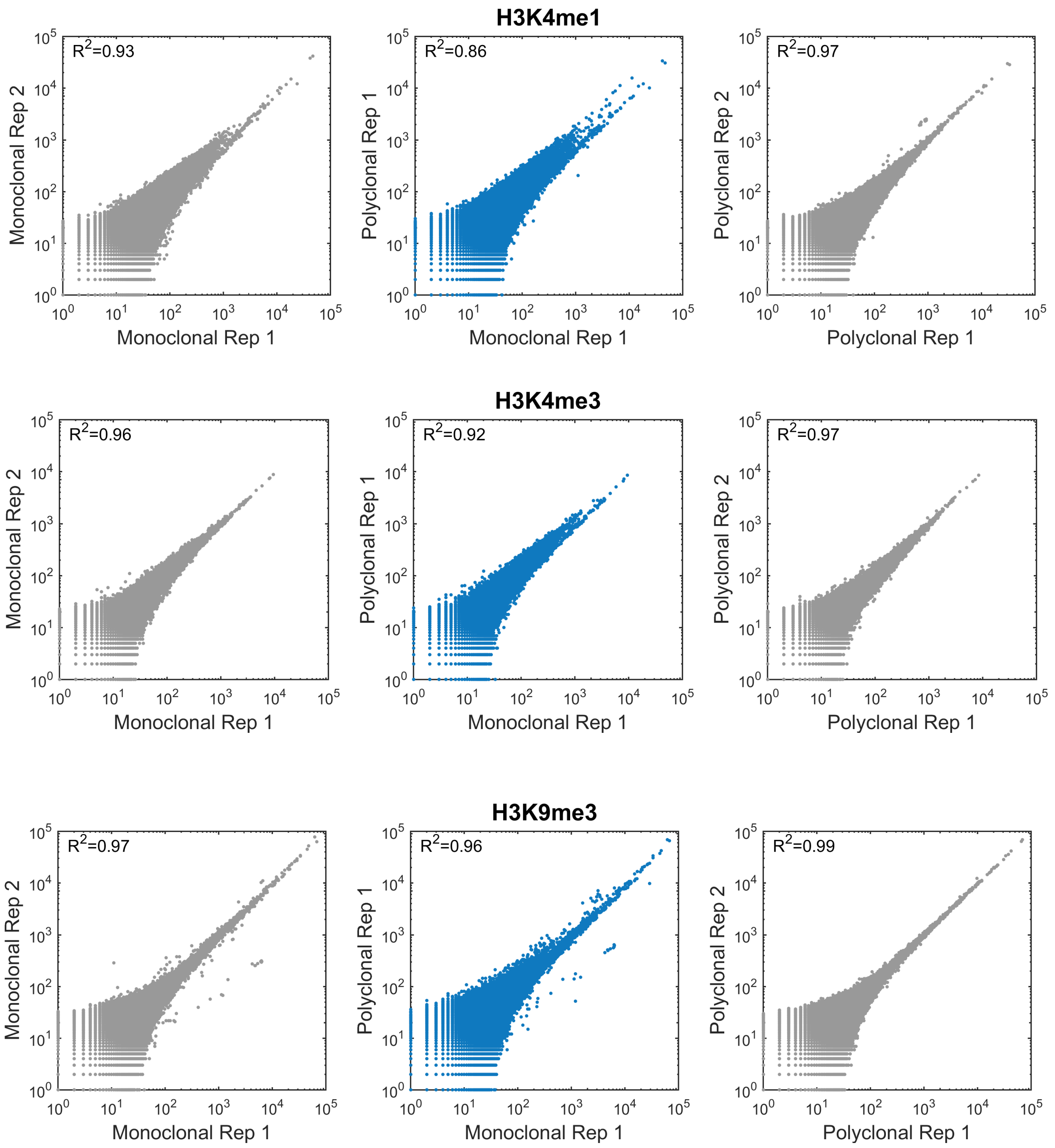
H3K4me1
R2=0.93
R2=0.86
R2=0.97
H3K4me3
R2=0.96
R2=0.92
R2=0.97
H3K9me3
R2=0.97
R2=0.96
R2=0.99
Monoclonal Rep 2
Monoclonal Rep 1
Polyclonal Rep 1
Polyclonal Rep 2
10^0 10^1 10^2 10^3 10^4 10^5

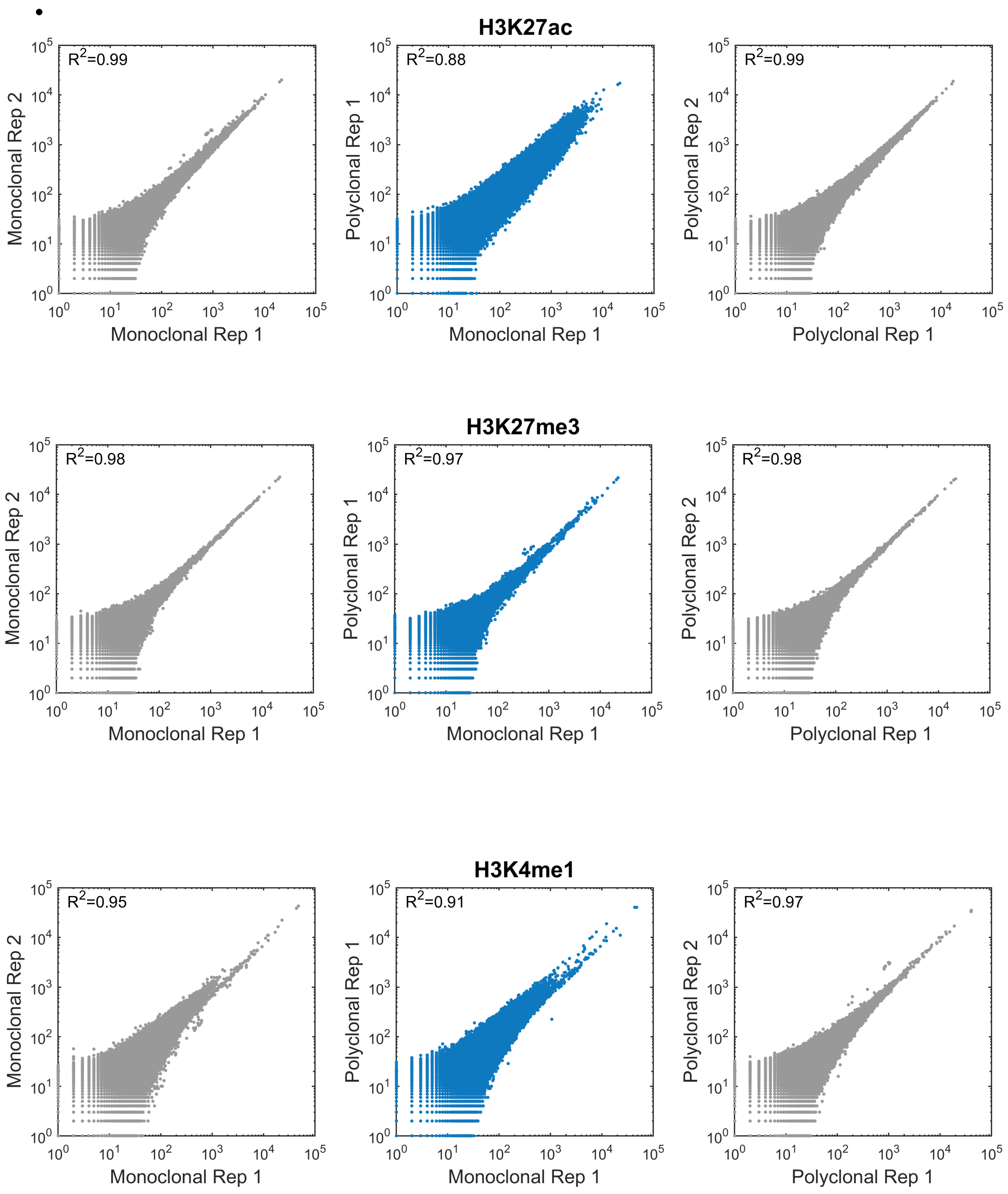
H3K27ac
R2=0.99
Monoclonal Rep 2
Monoclonal Rep 1
R2=0.88
Polyclonal Rep 1
Monoclonal Rep 1
R2=0.99
Polyclonal Rep 2
Polyclonal Rep 1
H3K27me3
R2=0.98
Monoclonal Rep 2
Monoclonal Rep 1
R2=0.97
Polyclonal Rep 1
Monoclonal Rep 1
R2=0.98
Polyclonal Rep 2
Polyclonal Rep 1
H3K4me1
R2=0.95
Monoclonal Rep 2
Monoclonal Rep 1
R2=0.91
Polyclonal Rep 1
Monoclonal Rep 1
R2=0.97
Polyclonal Rep 2
Polyclonal Rep 1

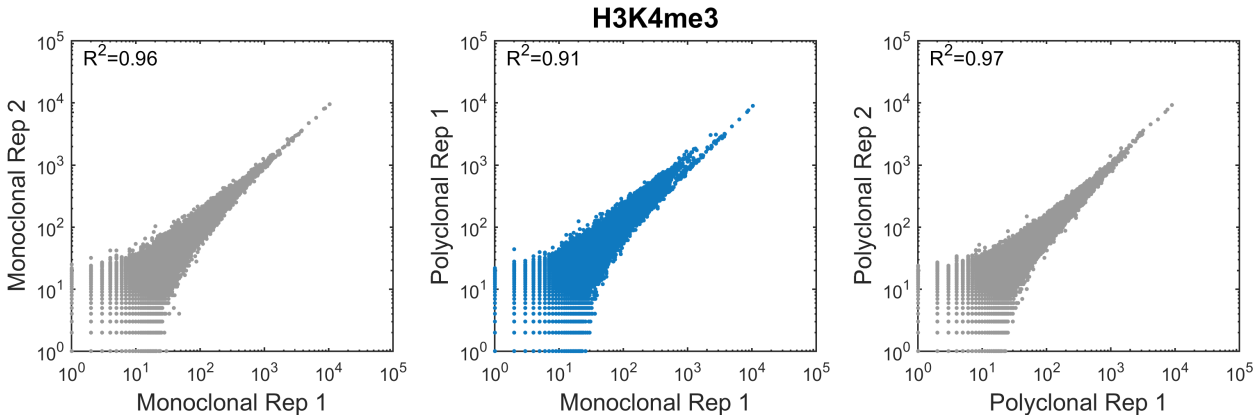
H3K4me3
R2=0.96
R2=0.91
R2=0.97
Monoclonal Rep 2
Polyclonal Rep 1
Polyclonal Rep 2
Monoclonal Rep 1
Monoclonal Rep 1
Polyclonal Rep 1

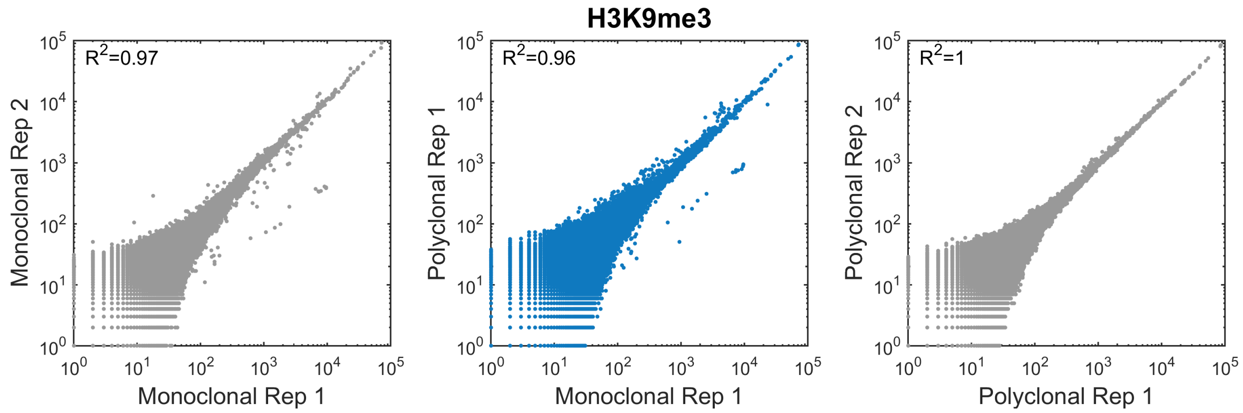
H3K9me3
R2=0.97
R2=0.96
R2=1
Monoclonal Rep 2
Polyclonal Rep 1
Polyclonal Rep 2
Monoclonal Rep 1
Monoclonal Rep 1
Polyclonal Rep 1

Figure S6.

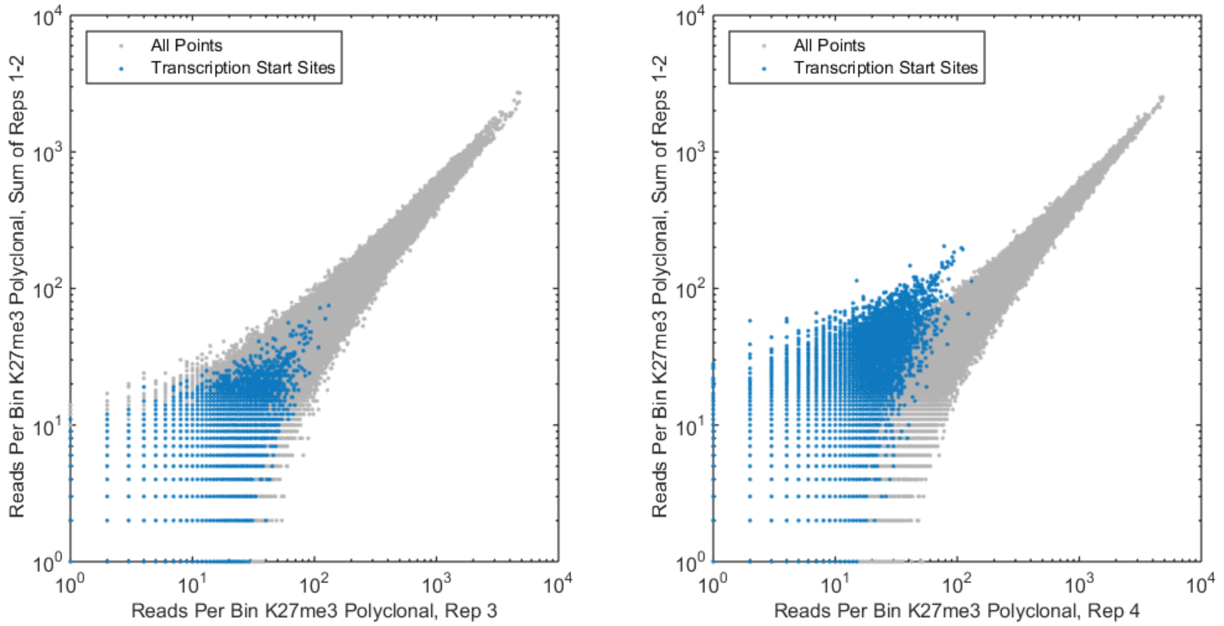

All Points
Transcription Start Sites
Reads Per Bin K27me3 Polyclonal, Sum of Reps 1-2
Reads Per Bin K27me3 Polyclonal, Rep 3
All Points
Transcription Start Sites
Reads Per Bin K27me3 Polyclonal, Sum of Reps 1-2
Reads Per Bin K27me3 Polyclonal, Rep 4

Figure S7.

Antibody: H3-K4me1
Vendor: Active Motif
Part number: 39297
Lot number: 1714002

Test Date: 4-1-2016
User: RI

Primary antibody:
Primary ab dilution: 1:20,000
Incubation: O/N at 4C

Secondary antibody:
Vendor: Active Motif
Part number: 100612
Lot number: 6012051
Concentration: 1:2500
Incubation: 1 hour at room temperature

Protocol:
- immerse array in 4 mL of blocking solution containing (TBST containing 5% non-fat dried milk)
- incubate on a rotator for 4 hours at room temperature
- carefully pour off buffer
- perform a quick rinse (30 seconds) with TBST buffer
- wash 3 x 5 minutes in TBST
- dilute primary antibody in 4 mL of Blocking solution
- incubate on a rotator overnight at 4°C
- carefully pour off buffer
- perform a quick rinse (30 seconds) with TBST buffer
- wash 3 x 5 minutes in TBST
- dilute secondary antibody in 4 mL of Blocking solution
- incubate on a rotator for 1 hour at room temperature
- carefully pour off buffer
- perform a quick rinse (30 seconds) with TBST buffer
- wash 3 x 5 minutes in TBST
- detect using FlourChemQ

Reactivity of test antibody with control peptides and singly modified peptides
Graph is scaled so that the intended primary reactivity is set to 1

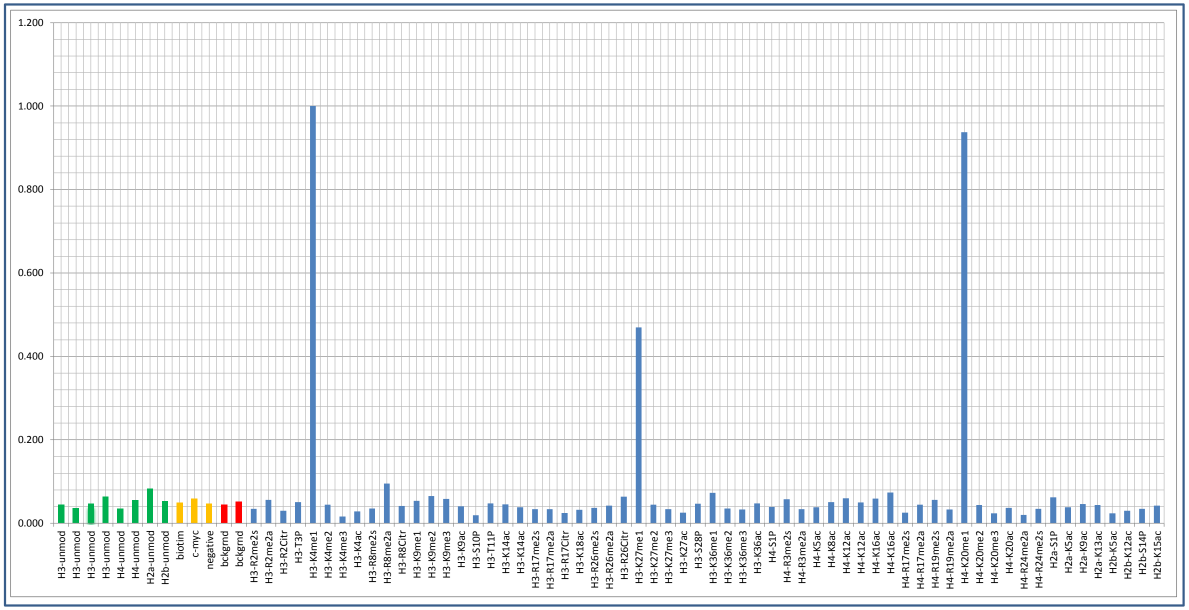

4-1-2016 - H3-K4me1 Active Motif #39297 Lot 1714002; image: 2013-05-24 14hr 26min_Exposure_2_0sec_crop-1_resize_data

Cross inhibition of reactivity by aother modifications of the same peptide
Graph is scaled so that saturation of signal = 1

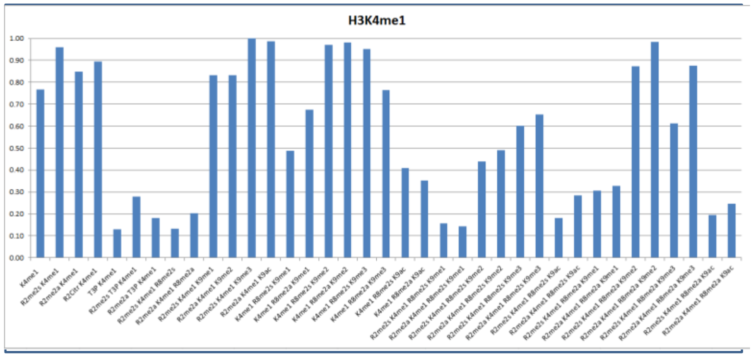

Gridded image of the array

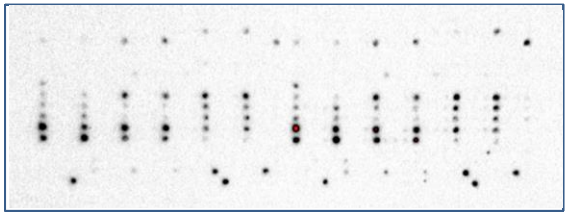

Supplement: Supplementary file 1 — Additional file 1: Figure S1. Reads aligning to annotated open and closed chromatin. Figure S2. Read coverage across the genome. Figure S3. Saturation curves. Figure S4. Mapping of peaks and reads to canonical chromatin regions. Figure S5. Correlation between monoclonal and polyclonal antibodies across the genome. Figure S6. Experimental quality control. Figure S7. Validation of the polyclonal antibody targeting H3K4me1 by peptide array. [file 13072_2016_100_MOESM1_ESM.pdf]
