# Supplementary material for: Systematic comparison of monoclonal versus polyclonal antibodies for mapping histone modifications by ChIP-seq
Source: Epigenetics Chromatin. 2016 Nov 4;9:49. doi: 10.1186/s13072-016-0100-6 (PMC5097419; doi:10.1186/s13072-016-0100-6)
Supplement: Supplementary file 2 — Additional file 2: Table S1. Datasets summary. [file 13072_2016_100_MOESM2_ESM.pdf]

Table S1. Datasets summary.

A.

Cell Type	Antibody	Replicate	Reads	Pairs
GM12878	H3K4me1	Monoclonal Rep 1	83,100,486	41,550,243
		Monoclonal Rep 2	99,255,196	49,627,598
	H3K4me3	Monoclonal Lot 8 Rep 1	99,255,196	78,227,985
		Monoclonal Lot 8 Rep 2	156,455,970	42,980,622
		Monoclonal Lot 9 Rep 1	85,961,244	33,912,720
		Monoclonal Lot 9 Rep 2	67,825,440	36,033,346
	H3K9me3	Monoclonal Rep 1	143,527,964	71,763,982
		Monoclonal Rep 2	66,206,100	33,103,050
	H3K27ac	Monoclonal Rep 1	146,244,844	73,122,422
		Monoclonal Rep 2	156,042,348	78,021,174
	H3K27me3	Monoclonal Rep 1	157,892,610	78,946,305
		Monoclonal Rep 2	152,454,660	76,227,330
HeLa	H3K27ac	Monoclonal Rep 1	17,340,796	8,670,398
		Monoclonal Rep 2	16,389,304	8,194,652
		Polyclonal Rep 2	13,155,478	6,577,739
K562	H3K4me1	Monoclonal Rep 1	44,707,286	22,353,643
		Monoclonal Rep 2	19,370,594	9,685,297
		Polyclonal Rep 1	46,708,750	23,354,375
		Polyclonal Rep 2	77,858,414	38,929,207
	H3K4me3 – lot comparison	Monoclonal Lot 8 Rep 1	83,285,982	41,642,991
		Monoclonal Lot 8 Rep 2	69,829,160	34,914,580
		Monoclonal Lot 9 Rep 1	73,635,980	36,817,990
		Monoclonal Lot 9 Rep 2	68,671,138	34,335,569
	H3K4me3	Monoclonal Rep 1	10,769,082	5,384,541
		Monoclonal Rep 2	14,706,444	7,353,222
		Monoclonal Rep 2	14,864,810	7,432,405
		Monoclonal Rep 4	11,230,992	5,615,496
		Polyclonal Rep 1	13,184,088	6,592,044
		Polyclonal Rep 2	10,007,054	5,003,527
		Polyclonal Rep 3	11,968,378	5,984,189
		Polyclonal Rep 4	25,205,992	12,602,996
	H3K9me3	Monoclonal Rep 1	75,625,321	37,812,661
		Monoclonal Rep 2	37,480,051	18,740,026
		Monoclonal Rep 2	45,075,156	22,537,578
	H3K27ac	Monoclonal Rep 1	42,775,420	21,387,710
		Monoclonal Rep 2	72,589,124	36,294,562
		Polyclonal Rep 1	60,003,204	30,001,602
		Polyclonal Rep 2	28,926,788	14,463,394

	H3K27me3	Monoclonal Rep 1	19,964,374	9,982,187
		Monoclonal Rep 2	24,909,516	12,454,758
		Monoclonal Rep 2	31,796,524	15,898,262
		Monoclonal Rep 4	25,983,780	12,991,890
		Polyclonal Rep 1	22,909,534	11,454,767
		Polyclonal Rep 2	28,428,020	14,214,010
		Polyclonal Rep 3	19,843,850	9,921,925
		Polyclonal Rep 4	37,134,412	18,567,206
	WCE	Rep 1	15,726,808	7,863,404
mES	H3K4me1	Monoclonal Rep 1	76,330,698	38,165,349
		Monoclonal Rep 2	98,191,020	49,095,510
	H3K4me3	Monoclonal Lot 8 Rep 1	81,525,630	40,762,815
		Monoclonal Lot 8 Rep 2	80,975,708	40,487,854
		Monoclonal Lot 9 Rep 1	72,554,992	36,277,496
		Monoclonal Lot 9 Rep 2	73,758,624	36,879,312
	H3K27ac	Monoclonal Rep 1	161,354,476	80,677,238
		Monoclonal Rep 2	161,371,680	80,685,840
	H3K27me3	Monoclonal Rep 1	75,925,468	37,962,734
		Monoclonal Rep 2	164,450,620	82,225,310

B.

		**Average Reads After Downsampling**	**Average Pairs After Downsampling**	**Pairs in Merged Dataset**
K562	H3K27ac	25,808,498	12,904,249	25,808,498
	H3K27me3	18,616,216	9,308,108	27,924,324
	H3K4me1	35,539,733	17,769,867	35,539,733
	H3K4me3	9,204,667	4,602,334	18,409,335
	H3K9me3	28,479,015	14,239,507	28,479,015
